# The nasal microbiota of two marine fish species: diversity, community structure, variability, and first insights into the impacts of climate change-related stressors

**DOI:** 10.1093/femsec/fiaf018

**Published:** 2025-02-17

**Authors:** Mishal Cohen-Rengifo, Cyril Noel, Elisabeth Ytteborg, Marie-Laure Bégout, Carlo C Lazado, Gwenaelle Le Blay, Dominique Hervio-Heath

**Affiliations:** Univ Brest, CNRS, IRD, Ifremer, LEMAR, IUEM, F-29280 Plouzané, France; IFREMER–PDG-IRSI-SEBIMER, F-29280 Plouzané, France; Nofima AS, The Norwegian Institute of Food, Fisheries and Aquaculture Research, 1433 Ås, Norway; IFREMER, Université Montpellier, CNRS, INRAE, IRD, MARBEC, F-34250 Palavas-les-Flots, France; Nofima AS, The Norwegian Institute of Food, Fisheries and Aquaculture Research, 1433 Ås, Norway; Univ Brest, CNRS, IRD, Ifremer, LEMAR, IUEM, F-29280 Plouzané, France; IFREMER, Univ Brest, CNRS, IRD, LEMAR, IUEM, F-29280 Plouzané, France

**Keywords:** nasal microbiota, bacterial diversity, olfactory rosette, metabarcoding, interindividual variability, climate change

## Abstract

Vertebrate nasal microbiota (NM) plays a key role regulating host olfaction, immunity, neuronal differentiation, and structuring the epithelium. However, little is known in fish. This study provides the first comprehensive analysis of the NM in two marine fish species, the European seabass and the Atlantic cod. Given its direct environmental exposure, fish NM is likely influenced by seawater fluctuations. We analysed the community structure, specificity regarding seawater, and interindividual variability of 32–38 fish reared under ambient conditions. Additionally, we conducted an experiment to investigate the influence of acidification and a simplified heatwave on cod NM (three fish per replicate). High-throughput 16S rRNA sequencing revealed species-specific NM communities at the genus-level with *Stenotrophomonas* and *Ralstonia* dominating seabass and cod NM, respectively. This suggests potential habitat- or physiology-related adaptations. The most abundant bacterial genera in seabass NM were also present in seawater, suggesting environmental acquisition. Alpha diversity was highest in Brest seabass NM and variability greatest in Tromsø cod NM. Simulated climate change-related scenarios did not significantly alter cod NM structure. We propose a minimum of 13 cod rosettes per replicate for future studies. This research establishes a foundation for understanding marine fish NM and its response to environmental changes.

## Teaser

Seabass and cod nasal microbiota showed distinct profiles from each other and from nonmarine fish, and varies with site in seabass but remains unaffected by climate change-related treatments in cod.

## Introduction

The olfactory organ of teleosts is composed of two olfactory rosettes, one situated in each nasal cavity. The rosette is lined with the olfactory epithelium (OE), which is a sheet of several types of cells arranged in two types of tissue: neuroepithelium and mucosal epithelium (Sepahi and Salinas 2016). The most important cells are the olfactory receptor neurons, whose olfactory cilia extend into a mucus layer and are in close contact with the external milieu (Purves et al. 2001). Odorants bind to olfactory cilia and trigger the process of olfactory transduction, which is mediated by the ionic properties of the mucus. The OE, in addition to being the first stage in the detection of odorants, serves as a gateway for water-borne physico-chemical particles and compounds, as well as a variety of microorganisms (Firestein 2001, Mori et al. 2005). The OE therefore plays a crucial role not only in olfaction but also in modulating immune responses to microorganisms as observed in various vertebrates including fish (Gomez et al. 2013, Tacchi et al. 2014, Li et al. 2017, Sepahi et al. 2019, Cohen-Rengifo et al. 2022, Thangaleela et al. 2022, Lazado et al. 2023, Vientos-Plotts et al. 2023).

Some microorganisms are able to colonize the host’s OE by living in association with the mucus layer. These symbiotic microorganisms are referred to as the nasal microbiota (NM). The NM has been described to play crucial role in various functions across terrestrial vertebrates including humans, such as protection against pathogens, aroma perception, and modulation of neuroimmune signals (Koskinen et al. 2018, Zeineldin et al. 2019, Di Stadio et al. 2020, Tai et al. 2021, Vientos-Plotts et al. 2023, Xi et al. 2024, Oladokun and Sharif 2024). A study on axenic mice revealed that the absence of NM causes a thinning of the ciliary layer of the olfactory receptor neurons, a reduction in cellular turn-over, and an increase in the intensity of neuronal electrical signals in response to odorants (François et al. 2016). Furthermore, the authors found decreased transcription of genes related to olfactory transduction actors and olfactory metabolizing enzymes, which might reduce the efficiency of odorant detection. The role of the NM in marine vertebrates is still poorly understood. Compared to other fish microbiota, such as those inhabiting the gut, but also the skin, and gills, fish NM has been relatively understudied (Lowrey et al. 2015, Piazzon et al. 2019, Rosado et al. 2021, Serra et al. 2021, Rangel et al. 2022). Furthermore, a significant knowledge gap exists regarding the NM of marine fish, as no prior studies have specifically investigated this microbial community.

The mucosal surfaces of all vertebrates have been subject to similar evolutionary pressures, leading to the development of conserved mechanisms involved in the sensory and immune systems in both terrestrial and aquatic animals (Tacchi et al. 2014). This is evident in the conserved nature of the nasal immune systems, encompassing both the nasopharynx-associated lymphoid tissue and the innate immune system, which notably rely on a large family of pattern recognition receptors and microbe-associated molecular pattern, across terrestrial and aquatic animals (Zhang et al. 2010, Tacchi et al. 2014, Sepahi and Salinas 2016, Li et al. 2017). Indeed, in axenic mice and zebrafish, structural changes, such as defective pseudo-stratification of the OE have also been observed (Casadei et al. 2019). Colonization of these animals by commensal bacteria resulted in transcriptional regulation of genes involved in olfactory organs. The NM was also observed to promote *in vitro* differentiation of odora cells in zebrafish (Casadei et al. 2019) and is likely to participate in mucosal immunity by coordinating host immune functions (Tai et al. 2021, Yu et al. 2021).

Fish mucosal surfaces are constantly challenged by the aquatic environment, and water is a major driver of fish microbiome composition. This interplay between the environment, the microbiome, and mucosal immunity is crucial for fish health (Morshed and Tsung-Han Lee 2023). Under the ongoing climatic changes, fish are vulnerable to a variety of anthropogenic pressures such as ocean warming, acidification, heatwaves, and hypoxia, among others (Hutchins and Fu 2017). Cohen-Rengifo et al. (2022), exposed seabass to transgenerational exposure to acidification (−0.4 pH units) and a subsequent viral challenge. Compared to fish from the ambient control group (pH 8.1), acidified individuals showed significant upregulation of genes involved in pathogen recognition and odour transduction programs within the OE. This led to improved survival rates following viral infection. Since the NM is surrounded by environmental seawater, changes in the biological and physico-chemical characteristics of seawater due to climate change might alter the NM composition and its functional contribution to the host (Lazado et al. 2023, Morshed and Tsung-Han Lee 2023). However, our understanding of these interactions under changing environmental conditions and their impact on fish immunity remains limited.

Studies addressing the impacts of climate change on marine fish have mainly focused on gut and skin microbiota, and revealed a distinct dysbiosis in response to ocean acidification or warming (Fonseca et al. 2019, Ghosh et al. 2022). For instance, ocean acidification (−0.4 pH units with respect to the control at pH 8.1) did not impact alpha diversity of gut microbiota in sea bream, but beta diversity revealed changes in phyla abundance with the phylum *Firmicutes* being absent in the acidified group (Fonseca et al. 2019). In contrast, exposure to simulated heatwaves (+5°C and +9°C above control temperatures of 24°C) for 3 months resulted in increased *Firmicutes* abundance across skin, gill, and gut microbiota. Concurrently, a reduction in microbial diversity was observed in all compartments, which correlated with a decrease in growth performance (Sánchez-Cueto et al. 2023). Many of the most common bacterial genera found in seawater and fish gut microbiota (e.g. *Pseudomonas* spp., *Vibrio* spp., *Enterobacter* spp., and *Tenacibaculum* spp.) contain opportunistic pathogen species, which could potentially induce host’s health problems if seawater conditions become favourable for their growth (Vatsos 2017). In light of the established role of the NM and the OE on host olfactory and immune transcriptional programmes, and considering the potential environmental impacts on NM composition, research in marine fish NM is necessary.

European seabass *Dicentrarchus labrax* and the Atlantic cod *Gadus morhua* are ecologically and commercially important marine fish species found across the Northeast Atlantic, with seabass extending into the Mediterranean and cod extending into the Northwest Atlantic and the Baltic Sea (Kijewska et al. 2016, FAO 2024, NOAA Fisheries 2024). Both are top predators exhibiting opportunistic feeding behaviours with diets depending on life cycle and prey availability (FAO 2024, NOAA Fisheries 2024). Seabass typically prefers coastal areas and estuaries although some venture further offshore (FAO 2024). In contrast, cod inhabit a wider range of habitats, including brackish waters (Kijewska et al. 2016, NOAA Fisheries 2024). Their economic importance as major fishery targets in Europe justifies the need for close monitoring and the adoption of sustainable management practices to address the risk of overfishing. France leads in seabass aquaculture, while cod fisheries are a cultural and economic cornerstone in Norway, requiring strict management alongside recent EU agreements (FAO 2024). In addition, their well-established captive breeding techniques for the entire life cycle make them relevant laboratory models that allow researchers to gain a thorough understanding of the life history of the breeders across all stages of development up to adulthood.

This study provides the first baseline information on the NM of two marine fish species (the European seabass *D. labrax* and the Atlantic cod *G. morhua*) hatched in captivity, while also providing new insights into the impact of climate change-related stressors on cod NM. The study employed two main approaches. First, it conducted a comparative analysis of the NM between seabass from Brest and Palavas, France, and cod from Tromsø, Norway, under ambient conditions. This analysis focused on the NM community structure, specificity regarding seawater and interindividual variability. Secondly, cods from Tromsø were exposed to four simulated climate change-related treatments: control, acidified, simplified heatwave, and combined acidified and simplified heatwave. This latter approach investigated potential changes in the structure of cod NM induced by these climate change-related conditions.

## Material and methods

Fish husbandry and manipulation followed the ethical standards of the institutions IFREMER and Nofima complying with recommendations of the Directive 2010/63/EU and the ARRIVE guidelines. The climate change-related simulation on cod was conducted with approval from the Norwegian Food Safety Authority (FOTS ID 29459).

### Husbandry of seabass

European seabass, *D. labrax*, hatched at IFREMER-Brest (France, 48° 21′ 33.9″ N 4° 33′ 33.4″ W) on 14 March 2021 or at IFREMER-Palavas (France, 43° 31′ 11.9″ N 3° 54′ 39.″ E) on 19 February 2021, and were reared under local ambient conditions following seasonality for 388 days for Brest seabass or 425 days for Palavas seabass. Seabass from either site were housed in a single rearing tank. Seabass were used for the species comparison analysis and were fed *ad libitum* with Neo Start Loops 3 and 4 for Brest and Palavas seabass (Le Gouessant, France), respectively.

Brest rearing operated under a flow-through system where seawater was pumped 500 m off the Dellec beach (Plouzané) at a depth of 20 m, and passed through a sand filter before being stored in a header tank, which supplies the water inlet of the rearing tank (500 l). Palavas rearing operated under a semiopen flow system, where seawater was pumped 300 m off the beach straight outside the research station at a depth of 2 m, and passed through a decantation tank and sand filters before being stored in a header tank, which supplies the water inlet of the rearing tank (1500 l).

### Husbandry of cod and climate change-related setup

Atlantic cod, *G. morhua*, were provided by the National Cod Breeding Program and hatched and reared until they were juveniles at the Centre of Marine Aquaculture (Kraknes, Norway, 69° 52′ 05.8″ N 18° 55′ 52.4″ E). They were then transported to the Tromsø Aquaculture Research Station (HiT Havbruksstasjonen i Tromsø, Bay of Inner Karkiva in the Kval channel) and held in quarantine for 3 weeks before they were transferred to the experimental tanks. Cod was used for both the species comparison analysis and the climate change-related simulation. Cod was reared under local ambient conditions following seasonality either for 324 days until they were dissected (on 27 June 2022 for the species comparison analysis) or for 292 days until they were transferred into other tanks (on 12 May 2022 for the climate change-related simulation). Cod was fed *ad libitum* with Amber Neptum (Skretting, Norway) pellets matching fish size and were subjected to a 24-h-light photoperiod.

For the climate change-related simulation, cods were evenly transferred into 12 rearing tanks (500 l, *n* = 60–70 fish) distributed in two rooms; one room for the control temperature treatments and the other one for the simplified heatwave treatments (Fig. S1A). Calibration of treatments took place for 16 days; ambient temperature (4.5°C) was increased by 1.5°C every 2 days until reaching the control temperature of 8°C, which corresponds to cod optimal for growth (Righton et al. 2010). A week after, pH was decreased from 8.1 by 0.1 pH units per day until reaching ∼7.7 (−0.4 ΔpH with respect to control pH). The experimental treatments were as follows: Control Treatment (CT: 8.1 pH units and 8°C), Acidified Treatment (AT: −0.4 ΔpH relative to the control pH and 8°C), Heatwave Treatment (HT: 8.1 pH units with a simplified heatwave regime of 8–16–8°C, described below), and Acidified and Heatwave Treatment (AHT: −0.4 ΔpH and the simplified heatwave regime). Our simplified heatwave regime consisted of five phases: a 6-day acclimation phase at 8°C followed by a 7-day heat increase of 1°C per day, then a 5-day heat plateau at 16°C, a 7-day heat decrease phase of 1°C per day, and a 6-day retro-acclimation phase at 8°C. Based on data from 2016 to 2022, an experimental temperature of 16°C was selected, reflecting the maximal summer water temperature recorded in Dønna, Norway (Ytteborg et al. 2023). The pH target of ∼7.7 aligns with the predicted 0.4 unit decrease in ocean surface pH for the Arctic Ocean under the IPCC RCP8.5 scenario. Each treatment was triplicated in one of the 12 rearing tanks (Fig. S1B). Fish were exposed to these treatments for 31 days (from 28 May to 27 June 2022).

Tromsø rearing operated under a flow-through system. Seawater was pumped at 200 m off the HiT coastline at a depth of 670 m and passed through rotation filters and a UV filter before being stored in a water tower. Seawater was initially delivered to a main header tank at ambient temperature. A portion of the seawater was diverted to a secondary header tank equipped with a heating system. From these header tanks, seawater was then distributed to the experimental rooms, with one for the control temperature treatments (CT and AT) and three for the heatwave treatments (HT and AHT). In the room, water was finally distributed to six individual rearing tanks (500 l) at a flow rate of 10 l min^−1^. Half of the rearing tanks of each room were also acidified for the acidified treatments (AT and AHT) (Fig. S1A). The acidification system consisted of a CO_2_ bottle connected to a model P flow tube rotameter (Alborg, USA) that injected CO_2_ into two mixing tanks at controlled constant flow of 30 ± 6 ml CO_2_ min^−1^. Mixed seawater from each mixing tank was then delivered into each of three acidified tanks per room. Each room had one mixing tank.

The pH (NIST scale) was measured daily in every tank, at least once a day (or more if pH adjustments were needed) using a Multi-Parameter Portable Meter Multi 3630 IDS equipped with a Sentix 9403 pH electrode (WTW, UK). Since heated water was stored in the same header tank, sea water temperature was measured daily in only one tank per room using a GMH 2710-K Probe thermometer (Greisinger, Germany). The percentage of dissolved oxygen (O_2_%) was measured weakly in every tank using a ProODO Optical Dissolved Oxygen Instrument (YSI, USA). Since salinity in the Bay of Inner Karkiva is stable, it was only measured at the beginning of the trial in every tank using the Multi-Parameter Portable Meter Multi 3630 IDS equipped WTW IDS digital conductivity cells TetraCon 925 (WTW). Seawater samples of 50 ml were collected weekly from every tank and stored at 4°C to measure total alkalinity. Alkalinity was measured by automated pH/Alkalinity titration using a Titroline 7000 run by the software Tirisoft 3.5 (Xylem Analytics, Germany). 50 ml of tank water were titrated with 0.1 N HCL. The carbonate system was then estimated using the software CO2SYS v2.1.

### Sampling

Samplings were carried out on 6 April 2022 for Brest seabass [age: 388 days posthatching—dph, body length (mean ± SD): 12.4 ± 1.3 cm], on 20 April 2022 for Palavas seabass (age: 425 dph, body length: 20.3 ± 1.9 cm), on 24 June 2022 for Tromsø cod reared under ambient conditions (age: 494–509 dph, body length: 27.9 ± 2.0 cm), and on 27 June 2022 for Tromsø cod reared under climate change-related conditions (age: 497–512, body length: 28.3 ± 1.7) (Table S1). Fish were haphazardly fished from their rearing tanks (one tank per site) without making any distinction between male or female individuals. We collected rosette samples from 32 Brest seabass, 34 Palavas seabass, and 38 Tromsø cods reared under ambient conditions. For the climate change-related experiment, three cods per tank for each treatment were fish. The order of sampling from each tank was randomized (Table S1).

Dissections were performed carefully in aseptic conditions to avoid any bacterial contamination of rosettes with skin microbiota. Rostrum skin was wiped to remove skin mucus and then carefully wiped with an antiseptic solution of povidone iodine (10%). The skin above the nostrils was removed with a one-shot cut using a sterile blade (Fig. S2). Both rosettes of each fish were removed with disinfected tweezers and stored in 1 ml of DNA shield (Zymo Research, USA) at room temperature for DNA extraction.

We collected two seawater samples from the water inlet of each rearing tank for each site and climate change-related treatments. One-litre sample of seawater from Brest or Palavas was collected in duplicate. Each sample was filtered using 0.22 µm polycarbonate Nucleopore Track-Etch membrane filters of Ø 47 mm (Whatman, UK). Filters were stored in 2 ml of DNA shield at room temperature.

### Bacterial DNA extraction

Rosettes and seawater samples stored in DNA shield as well as negative controls of extraction were centrifuged (10 min, 10 000 × *g* at room temperature—Sigma 1K15 Bioblock Scientific or Eppendorf Centrifuge 5424 R) and the supernatants were discarded. Seabass rosettes samples were incubated 90 min under agitation (300 r m^−1^ at 45°C) in a lysis buffer (238 µl) containing 27 µl of SDS 20%, 11 µl of Proteinase K (20 mg ml^−1^) and 200 µl of TNE buffer (Tris Base 1 M, NaCl 5 M and EDTA 0.5 M). In contrast, due to their larger size, two enzymatic lysis were performed for cod rosettes. First, cod rosette samples were incubated 90 min under agitation (300 r m^−1^ at 45 °C) in the lysis buffer (357 µl) consisting of 40.5 µl of SDS 20%, 16.5 µl of Proteinase K (20 mg ml^−1^) and 300 µl of TNE buffer. Then, after centrifugation (10 min, 10 000 × *g* at room temperature), the supernatants were collected in 1.5 ml Epperdorf tubes and placed on ice. For the second lysis of cod rosettes samples, pellets were incubated for 30 min under agitation (300 r m^−1^ at 45 °C) in a lysis buffer (178.5 µl) consisting of 20.25 µl of SDS 20%, 8.25 µl of Proteinase K (20 mg ml^−1^) and 150 µl of TNE buffer. Seawater samples were incubated 90 min (300 r m^−1^ at 45 °C) in a lysis buffer (594 µl) consisting of 67 µl of SDS 20%, 27 µl of Proteinase K (20 mg ml^−1^) and 500 µl of TNE buffer.

Following the different lysis process, all samples were centrifuged at 10 000 *× g* for 10 min at RT. For cod rosettes, the supernatants were collected and mixed in the same 1.5 ml Epperdorf tubes previously placed on ice. Then, 200 µl of each sample’s supernatant were transferred to a Lysing Matrix E tube and homogenized in a FastPrep-96^TM^ instrument (MP Biomedicals, USA) for 40 s at 6 m s^−1^ for seabass samples, or for 30 s at 800 r m^−1^ (roughly equivalent to 8.5–12.5 m s^−1^) for cod samples. Bacterial genomic DNA was extracted using the FastDNA Spin Kit for Soil (MP Biomedicals) according to the manufacturer’s instructions. DNA of rosette, seawater, and negative extraction control samples was eluted in 75 µl of molecular grade water and DNA concentration was estimated using a Qubit fluorometric system (Thermofisher Scientific, USA). DNA extracted from seawater samples from Tromsø did not meet the minimum quality requirements (quantity and purity) for further analyses and thus, comparison of cod NM and rearing seawater was not possible. DNA extracts were stored at −80°C prior to 16S rRNA gene amplicon library preparation and sequencing.

### 16S rRNA genes library preparation and MiSeq sequencing

All DNA extracts including negative extraction controls, were used as template for polymerase chain reaction (PCR) amplification of the hypervariable V3–V4 region of the 16S rRNA loci using the primer set PCR1F_460 (5′-ACG GRA GGC AGC AG-3′) and PCR1R_460 (5′-TAC CAG GGT ATC TAA TCC T-3′) (Boukerb et al. 2021). The amplicon length was 460 bp. PCR reactions were performed using a TECHNE TC-5000 PCR Thermal Cycler on a 25 µl reaction mixtures containing 0.38 µl of each primer (20 µM), 12.5 µl of Phusion^TM^ Plus PCR Master Mix 2X (ThermoScientific, USA), 8.75 µl of molecular grade water, and 3 µl of genomic DNA. PCR conditions were as follows: one predenaturation step at 98°C for 1 min followed by 30 cycles of denaturation at 98°C for 10 s, annealing at 60°C for 30 s, extension at 72°C for 20 s, and a final step of postelongation at 72°C for 5 min, followed by a forever-holding temperature of 4°C. PCR products quality and integrity were determined using electrophoresis on 1.5% agarose gel electrophoresis (2 h, 120 V, GelRed, Biotium, USA). Purified genomic DNA samples were loaded into a cartridge and sent to McGill University (Genome Quebec Innovation Centre, Montréal, QC, Canada) for barcode library generation and sequencing. The sequencing run was carried out using the Illumina MiSeq (PE300 10 M reads) method (2 × 250 paired-end for 250 bp raw read length).

### Bioinformatics and statistics

Bioinformatic analyses were performed using the open-source modular workflow SAMBA v4.0.1 (https://gitlab.ifremer.fr/bioinfo/workflows/samba; Standardized and Automated Metabarcoding Analyses workflow). First, a checking process was carried out through SAMBA in order to verify the integrity of sequencing raw data. Then, using QIIME 2 (Bolyen et al. 2019) and the Cutadapt plugin, PCR primers were removed with an overlap of 13 and an error rate of 0.1. Sequences without primers were excluded from further analysis. Using DADA2 (Callahan et al. 2016), amplicon sequence variants (ASVs) were inferred after filtering (trunQ = 2, FmaxEE = 2, RmaxEE = 2, and n_read_learn = 1 000 000), denoising (independent method), forward and reverse merging (no removal nor trimming of primers), and chimeras detection (consensus method). According to similarity, distribution and abundance profiles, ASVs sequences were then clustered to limit false-positive ASV (PCR bias, uncorrected sequencing error) using the dbOTU3 (Olesen et al. 2017) algorithm (genetic criterion: 0.1, abundance criterion: 10, and *P*-value criterion = .0005). Taxonomic assignment was performed using a Naïve Bayesian classification against the SILVA v138.1 database (updated in August 2020; Quast et al. 2013). A decontamination process was also performed with SAMBA using *microDecon* R package with respect to the negative controls of extraction.

Phyloseq objects for each project generated by SAMBA were uploaded into R-4.0.5 (R Core Team 2021) to perform all diversity and statistical analyses using home-made scripts using mainly the *phyloseq* and *vegan* R packages. All graphs were built using the *ggplot2* R package. An intersect analysis using the *UpSetR* R package was performed to evaluate the number of exclusive or overlapping ASVs at the phylum and genus levels. The analysis of composition of microbiomes (ANCOM) was performed to determine the differential abundance of microbial genera in terms of log fold change (LFC) (Mandal et al. 2015). Alpha diversity (within sample diversity) was addressed through the Observed Richness index and Shannon diversity index. For beta diversity analyses (between sample diversity), a cumulative sum scaling normalization was done on the data. Then, a principal coordinate analysis (PCoA) was carried out to visualize similarities between matrices computed according to four distance metrics: Bray–Curtis accounting for abundance, Jaccard accounting for presence/absence, Unifrac accounting for phylogeny, and Weighted Unifrac (Wunifrac) accounting for both abundance and phylogeny. Interindividual variability of rosette samples from fish reared either under ambient or climate change-related conditions was addressed through a Permutation Test for Homogeneity of Multivariate Dispersions (beta dispersion) based on comparisons of the dispersion distance from centroid (DispDist) using both Bray–Curtis and Wunifrac.

An Interindividual Variability Model was conceived to determine a range of samples needed to cover 95%–99% of the NM variability for each site. Wunifrac beta dispersion was selected for our predictive model because it is a more comprehensive measure of beta diversity than Bray–Curtis. A group of samples from two to the maximal number of samples (34 for Brest seabass, to 32 for Palavas seabass and to 38 for Tromsø cod) were randomly selected. The DispDist using Wunifrac was calculated for each group of samples. This calculation was permuted 1000 times and allowed to estimate the mean DispDist per group of samples (*n*). Then, the variation difference (DiffVar, in percentage) for each *n* was calculated as the percentage difference between the mean DispDist for the group with the maximal number of samples (mean DispDist_*max*_) and the mean DispDist for each n (mean DispDist_*n*_) divided by mean DispDist_*max*_ as follows:


\begin{eqnarray*}
\textit{DiffVa}{r_n}\,\,\left( \% \right) = \,\,\frac{{\left( {\left( {\textit{mean}\,\,\textit{DispDis}{t_{max}} - \textit{mean}\,\,\textit{DispDis}{t_n}} \right)*100\% } \right)}}{{\textit{mean}\,\,\textit{DispDis}{t_{max}}}}.
\end{eqnarray*}

The range of samples needed to cover 95%–99% of NM interindividual variability is when DiffVar situates between a maximal variation of ≤5% and a minimal variation of <1%.

FASTQ files are available in (accession numbers link will be available upon publication of this paper). All raw data and homemade scripts are available as Supplementary Information.

## Results

### Species comparison

#### Raw data analysis

Illumina sequencing of the 16S rRNA V3–V4 region yielded a total of 4862 596 demultiplexed sequences. Following a series of data cleaning steps, including the removal of low-quality reads, primer sequences and chimeras, and the clustering of ASVs, 3480 345 high-quality reads (72%) were obtained. After additional processing with microDecon (McKnight et al. 2019), a total of 2707 393 sequences (56%) remained suitable for downstream analysis.

#### Alpha diversity

**Figure 1.**
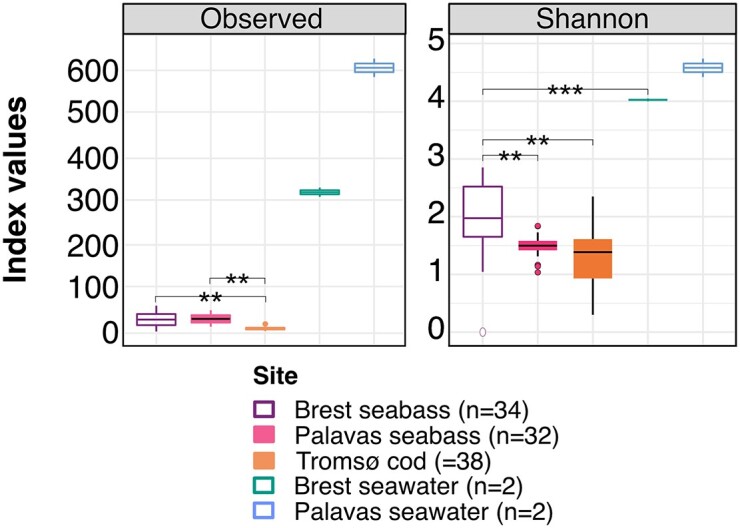
Alpha diversity indexes (within-sample diversity) of the NM in European seabass and Atlantic cod reared under ambient conditions, and of the bacterial community present seawater, as measured by the observed richness (A) and the Shannon diversity index (B). Boxes and dots are coloured by site. ** *P*-value = .001–.01; ***: *P*-value ≤ .001. Note: Tromsø seawater samples did not meet DNA quality requirements.

A total of 2202 ASVs were identified from the rosette samples. Among these, 979 ASVs originated from Brest seabass, 926 ASVs from Palavas seabass, and only 297 ASVs from Tromsø cod. Observed richness was significantly lower in Tromsø cod NM (8 ± 4, values are shown as mean ± SD hereinafter) compared to both Brest (29 ± 14, *p*-adj ≤ .001) and Palavas seabass NM (29 ± 10, *p*-adj ≤ .001), which were not different from each other (*p*-adj = 1) (Fig. 1A, Table S2). However, the Shannon diversity index revealed significantly higher diversity in Brest seabass NM (2.0 ± 0.6) compared to both Palavas seabass NM (1.5 ± 0.2; *p*-adj = .001) and Tromsø cod NM (1.3 ± 0.5; *p*-adj ≤ .001). There was no difference in Shannon diversity between the Palavas seabass NM and the Tromsø cod NM (Fig. 1B, Table S2).

With respect to seawater, a total of 1853 ASVs were identified. Of these, 641 ASVs originated from Brest, while 1212 ASVs came from Palavas. As expected, seawater samples were highly rich (Brest: 15 ± 4.0, Palavas: 30 ± 4.6) and diversified (Brest: 4.0 ± 0.04, Palavas: 4.6 ± 0.2). There was no significant difference between Brest and Palavas in terms of observed richness (*p*-adj = .3) and Shannon diversity (*p*-adj = 1). As observed for the NM, seawater richness did not show any statistical differences in either Brest (*p*-adj = .3) or Palavas (*p*-adj = .4). However, seawater diversity was statistically higher than NM diversity in Brest (*p*-adj ≤ .001) but not in Palavas (*p*-adj = .3).

#### Beta diversity

**Figure 2.**
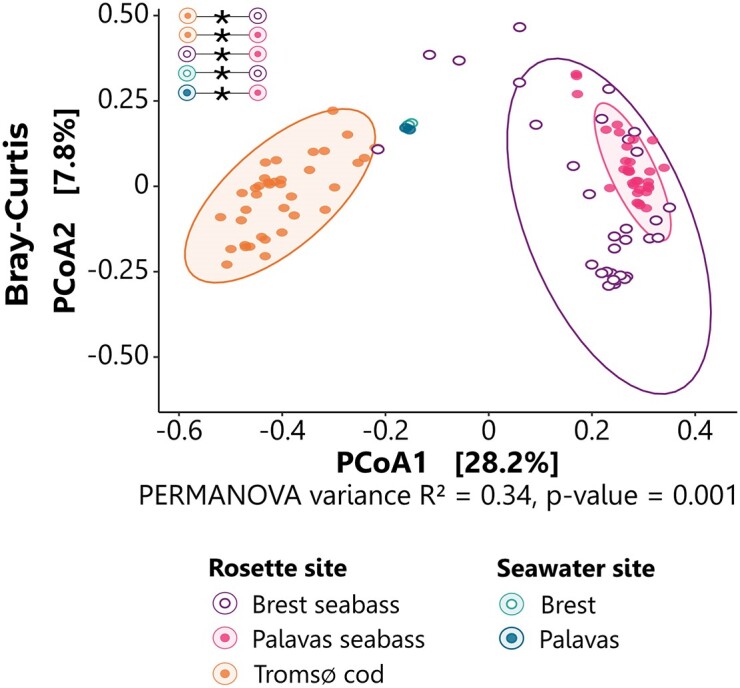
PCoA illustrating beta diversity of the NM in European seabass and Atlantic cod reared under ambient conditions and of the seawater bacterial community according to site. PCoAs were computed on Bray–Curtis dissimilarity matrices. Each dot represents a sample and is coloured by rosette or seawater site. Contrasts’ significances are shown as * if *p*-adj ≤ .05.

Dissimilarities of the bacterial community structure was estimated using Bray–Curtis and Wunifrac distances metrics. Bray–Curtis-based PERMANOVA revealed that 34% of the variance was explained by dissimilarities (p_Bray–Curtis_ = 0.001). Bray–Curtis-based PCoA analysis revealed that the seabass NM were very similar, independently of site (Brest or Palavas), whereas they were clearly different from the Cod NM (Tromsø) and seawater samples (Brest and Palavas) (Fig. 2). Wunifrac-based PERMANOVA showed only 13% of the variance and was explained by dissimilarities encompassing both taxa abundance and phylogeny (p_Wunifrac_ = 0.032). Significant differences were only observed between NM and seawater for both Brest (*p*-adj = .03) and Palavas (*p*-adj = .02) (Tables S3, see also for Jaccard and Unifrac results).

#### Taxonomic profiles

Metabarcoding analyses of the olfactory rosette in captive fish and their rearing seawater identified members of the bacteria domain, exclusively. The NM comprised a total of 11 phyla in Brest seabass, 13 phyla in Palavas seabass, and 10 phyla in Tromsø cod, which were respectively distributed in 106, 97, and 79 genera. In contrast, the bacterial community in seawater was much richer and comprised a total of 24 phyla in Brest and 28 phyla in Palavas, which were respectively distributed in 199 and 248 genera. It is noteworthy that phylum and genus names shown below correspond to the classification based on the SILVA v138.1 database (Quast et al. 2013). However, correct current phylum names are shown in parenthesis according to The Genome Taxonomy Database (GTDB; Chaumeil et al. 2022) and the List of Prokaryotic names with Standing in Nomenclature (LPSN; Meier-Kolthoff et al. 2022).

**Figure 3.**
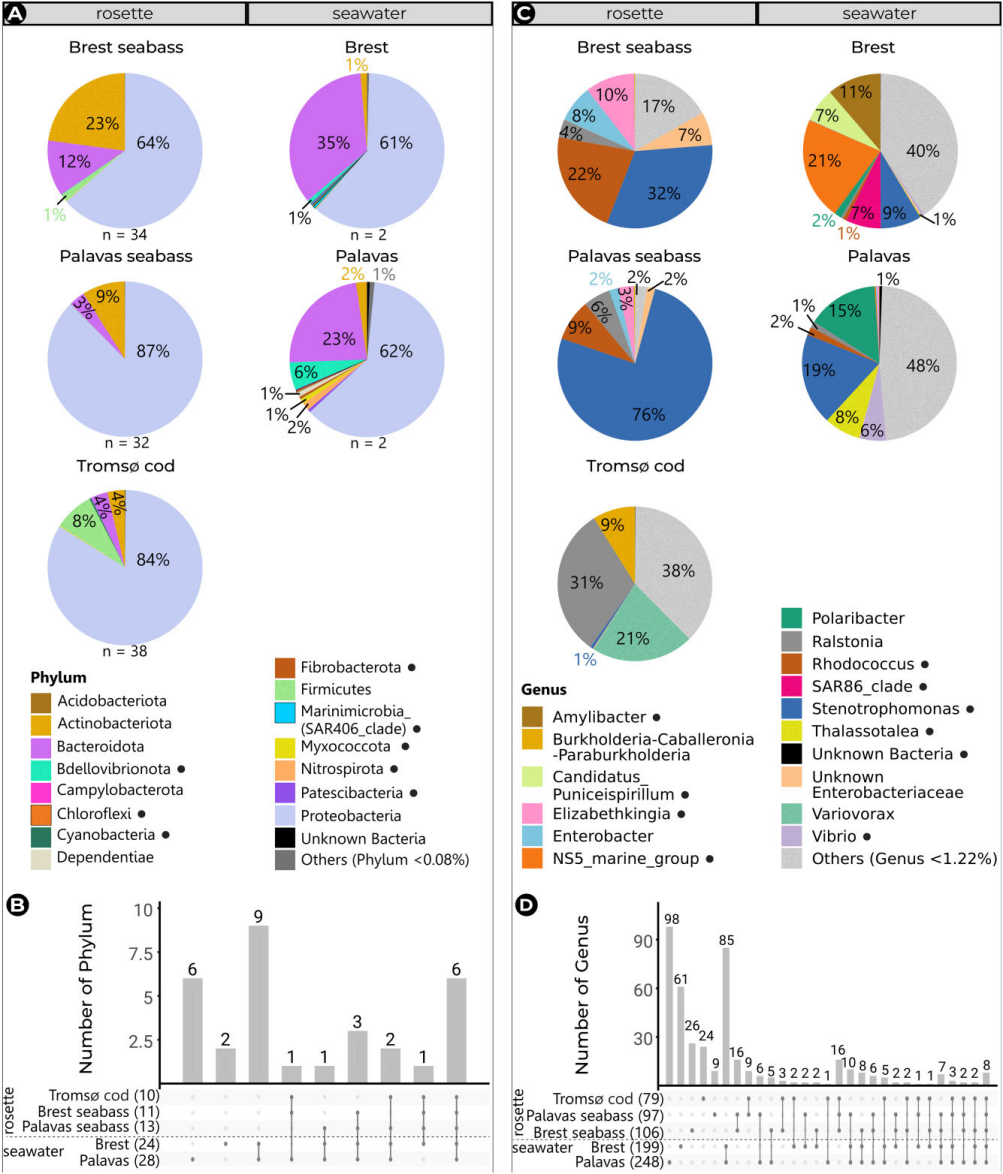
Taxonomic profile of the NM in European seabass and Atlantic cod reared under ambient conditions and of the seawater bacterial community according to site. Proportional distribution of the 15 most predominant bacterial phyla (A) and genera (C) including the number of exclusive and common phyla (B) or genera (D). The total number of phyla or genera per condition is in parenthesis. ●: most predominant taxa whose abundances significantly differed between seawater sites.

In both seabass and cod NM, four phyla were abundantly represented under all conditions, with a majority of *Proteobacteria* (currently classified as *Pseudomonadota*), followed by *Actinobacteriota* (currently *Actinomycetota*), *Bacteroidota*, and *Firmicutes* (currently *Bacillota*) (Fig. 3A). No phylum was exclusively present in any site (Fig. 3B). The relative abundances of *Proteobacteria* and *Firmicutes* did not vary significantly between sites. However, *Actinobacteriota* and *Bacteroidota* were ~4 LFC less abundant in cod than in seabass (*p*-adj ≤ .001; Fig. S2A). In seabass NM, the abundance of the most predominant genera was largely similar between Brest and Palavas and belonged to *Proteobacteria* (*Ralstonia, Stenotrophomonas, Thalassotalea*, and unknown *Enterobacteriaceae*), *Actinobacteriota* (*Rhodococcus*), and *Bacteroidota* (*Elizabethkingia*). Only the genera *Stenotrophomonas* (LFC = 5.4, *p*-adj_ANCOM_ ≤ .001) and *Burkholderia–Caballeronia–Paraburkholderia* (LFC = 2.2, *p*-adj_ANCOM_ = .008) were significantly more abundant in Palavas (76% and 0.2%) compared to Brest (32% and 0.3%) (Fig. S2A). In contrast, cod NM exhibited a markedly different profile with all of the most abundant genera belonging to *Proteobacteria* (*Ralstonia* at 31%, *Variovorax* at 21%, and *Burkholderia–Caballeronia–Paraburkholderia* at 9%), while 38% of other genera were present at <1%.

Differences between seabass and cod NM observed in the Bray–Curtis PCoA were mainly due to abundance rather than to phylogeny. Indeed, out of a total of 142 identified genera found in the NM, 16 were consistently found in all NM regardless of site, 16 others were exclusively found in seabass NM (Fig. 3C and D), whereas 79 were exclusively found in cod NM. The genera *Stenotrophomonas* and *Rhodococcus* were between 3 and 9 LFC less abundant—or absent—in Tromsø cod (1% and 0%) than in Brest seabass (32% and 22%) and Palavas seabass (76% and 9%) (*p*-adj_ANCOM_ ≤ .001; Fig. S2A). In contrast, *Burkholderia–Caballeronia–Paraburkholderia* was 5 LFC more abundant in Tromsø cod than in Brest seabass (*p*-adj ≤ .001), whereas *Variovorax* was only present in Tromsø cod at ~8 LFC higher abundance than in Brest or Palavas seabass (*p*-adj_ANCOM_ ≤ .001) (Fig. S2A).

In seawater, most phyla were found in both sites, with *Proteobacteria* and *Bacteroidota* dominating in both Brest (61% and 35%) and Palavas (62% and 23%) at statistically similar abundances. Following these, *Bdellovibrionota* (currently *Pseudomonadota*; 0.8% versus 5.7%), *Actinobacteriota* (1.4% versus 2.2%), and *Firmicutes* (0.007% versus 0.1%) were found at much lower abundances. The phyla *Cyanobacteria* (currently *Cyanobacteriota*) and *Marinimicrobia* (SAR406_clade) (currently *Pseudomonadota*) were only found in Brest seawater, whereas the phylum *Chloroflexi* (currently *Chloroflexota*) was only found in Palavas seawater. At the genus level, the two waters greatly differed. Among a total of 236 identified genera found in seawater, 85 genera were present in seawater from both sites, 98 genera were exclusive to Brest seawater, while 61 genera were found exclusively in Palavas seawater (Fig. 3D). The proportion of rare genera in both Brest and Palavas seawater was high, with 40% and 48%, respectively (Fig. 3C). The most predominant genera belonged to *Proteobacteria* (*Amylibacter, Burkholderia–Caballeronia–Paraburkholderia, Candidatus_Puniceispirillum, Enterobacter, Ralstonia*, SAR86_clade, *Stenotrophomonas, Thalassotalea*, unknown *Enterobacteriaceae, Variovorax*, and *Vibrio*), *Actinobacteriota* (*Rhodococcus*), and *Bacteroidota* (*Elizabethkingia, NS5_marine_group*, and *Polaribacter*) (Fig. 3C).

If we look at the differences in taxa abundance between seawater sites, there were 118 genera whose abundances significantly differed between sites. Among these, 10 genera were part of the most predominant genera and are indicated with a black circle in Fig. 3(C). For instance, *Amylibacter* (0.01%, LFC = −7.2, *p*-adj_ANCOM_ ≤ .001) and NS5_marine_group (0.3%, LFC = −4.5, *p*-adj ≤ .001) were significantly less abundant in Palavas than in Brest, whereas *Thalassotalea* (8%, LFC = 3.8, *p*-adj_ANCOM_ ≤ .001) and *Vibrio* (6%, LFC = 2.1, *p*-adj_ANCOM_ = .01) were significantly more abundant in Palavas than in Brest (Fig. S2B).

Regarding the taxa present in both seabass NM and seawater, five phyla were common to all five conditions: *Actinobacteriota, Bacteroidota, Firmicutes, Myxococcota* (currently *Pseudomonadota*), *Proteobacteria*, and Unknown Bacteria (Fig. 3A and B). Additionally, *Acidobacteriota* and *Bdellovibrionota* were common to seabass rosettes and their rearing seawater (Fig. 3A and B). All phyla found in seabass NM were also present in seawater. *Bdellovibrionota, Myxococcota*, and Unknown Bacteria were present in significantly higher abundance in seawater than in seabass for both Brest (*Bdellovibrionota*: LFC = 5.7, *p*-adj_ANCOM_ ≤ .001; *Myxococcota*: LFC = 3.8, *p*-adj_ANCOM_ = .04; unknown Bacteria: LFC = 3.3, *p*-adj_ANCOM_ = .003), and Palavas (*Bdellovibrionota*: LFC = 7.4, *p*-adj_ANCOM_ ≤ .001; *Myxococcota*: LFC = 5.8, *p*-adj_ANCOM_ ≤ .001; unknown Bacteria: LFC = 5.2, *p*-adj_ANCOM_ ≤ .001) (Fig. 3C). At the genus level, five (*Enterobacter, Ralstonia, Rhodococcus, Stenotrophomonas*, and Unknown *Enterobacteriaceae*) of the most predominant genera were common to all rosette and seawater samples (Fig. 3C).

If we compare seabass NM and their rearing seawater within each site, there were more genera showing a significantly different abundance between seawater and NM in Palavas than in Brest (Fig. S2C). For instance, in Palavas, *Polaribacter, Thalassotalea*, and *Vibrio* were ~7 LFC (*p*-adj_ANCOM_ ≤ .001) more abundant in seawater (15%, 8%, and 6%) than in the NM (0.1%, 0.03%, and 0.07%), whereas Unknown *Enterobacteriaceae* was 3 LFC less abundant in seawater (0.1%) than in the NM (2%) (Fig. 3C, Fig. S2C). In Brest, NS5_marine_group was 9 LFC (*p*-adj_ANCOM_ ≤ .001) more abundant in seawater (21%) than in the NM (0.03%) (Fig. 3C, Fig. S2C). Moreover, Fig. 3(D) shows that 35 genera were exclusively present in seabass rosettes, 16 of which were shared by fish from Brest and Palavas and absent from seawater.

The NM showed high variability within sites, particularly in Brest seabass and Tromsø cod. For instance, *Stenotrophomonas* was absent in half (50%) of the Brest seabass samples, but present in the other half at varying abundances (ranging from 12% to 100%) (Fig. S3A). *Ralstonia* was detected in only 42% of Tromsø cod samples, with abundances varying considerably between 3% and 92%, while *Variovorax* was present in 84% of the samples at abundances ranging from 1% to 69%. (Fig. S3C). In contrast, all Palavas seabass samples contained *Stenotrophomonas*, with abundances ranging from 64% to 92% (Fig. S3B). This high variability made it challenging to establish a core NM since only a few genera were present in 100% of the samples from each site. Applying a threshold of 80% presence, the core NM of seabass included *Citrobacter, Elizabethkingia, Enterobacter, Rhodococcus*, and *Stenotrophomonas*. In contrast, the core NM of Tromsø cod was minimal, consisting solely of *Burkholderia–Caballeronia–Paraburkholderia* and *Variovorax*.

#### Beta dispersion and interindividual variability

The interindividual variability of the NM, based on Bray–Curtis beta dispersion, varied with site (Permutation test F = 175.5; *p*-adj ≤ .001). It was significantly higher in Tromsø cod (DispDist = 0.6) compared to Brest seabass (DispDist = 0.5), and significantly lower in Palavas seabass (DispDist = 0.1) compared to the other conditions (Table S4). Similarly, interindividual variability of the NM based on Wunifrac beta dispersion (Permutation test F = 14.7; *p*-adj ≤ .001) was higher in Brest seabass (DispDist = 0.3) than in Palavas seabass (DispDist = 0.01) or Tromsø cod (DispDist = 0.01) (Table S4).

**Figure 4.**
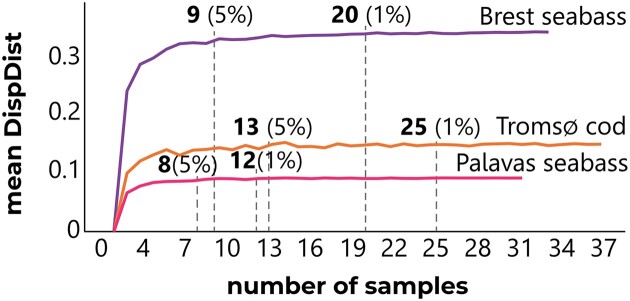
Interindividual Variability Model based on the mean dispersion distance from centroid (DispDist, computed with weighted Unifrac) per number of samples for the NM in European seabass and Atlantic cod reared under ambient conditions according to site. Bold numbers above the coloured curves represent the number of samples at which the difference variation (DiffVar) ranges between 1% (right) and 5% (left).

Our Interindividual Variability Model based on Wunifrac beta dispersion, revealed that the mean DispDist reached a plateau at a lower number of samples for Palavas seabass and a higher number of samples for Tromsø cod (Fig. 4, Table S5). This is valid to cover both 95% and 99% of the variability. The minimum number of samples per tank required to cover 95% of the variability would be 8 for Palavas seabass, 9 for Brest seabass, or 13 for Tromsø cod.

### Climate change-related simulation on cod NM

#### Seawater parameters

The present study successfully performed the simulated target treatments (CT: Control Treatment, AT: Acidified Treatment, HT: Heatwave Treatment, and AHT: Acidified and Heatwave Treatment) throughout the 31-day climate change-related simulation. The standard deviations (SD) of daily pH values were similar to what is typically observed in acidification studies (raw data link available). The average pH values (± SD) during the experiment were: 8.082 ± 0.004 in CT, 7.700 ± 0.009 in AT, 8.056 ± 0.009 in HT, and 7.697 ± 0.013 in AHT. The mean ΔpH between the ambient pH treatments and the low pH treatment was 0.38 ± 0.04. Seawater and carbonate system parameters are available in Table S6.

#### Raw data analysis on cod NM

Illumina sequencing of the 16S rRNA V3–V4 region from cod rosette samples yielded a total of 1351 573 demultiplexed sequences. Following a series of data cleaning steps, which involved the removal of low-quality reads, primer sequences, and chimeras, and the clustering of ASVs, 1010 007 high-quality reads (76%) were obtained. After additional processing with microDecon, a total of 476 474 sequences (35%) remained suitable for downstream analysis. Ultimately, only samples containing >800 sequences were included in the analysis.

#### Alpha diversity

**Figure 5.**
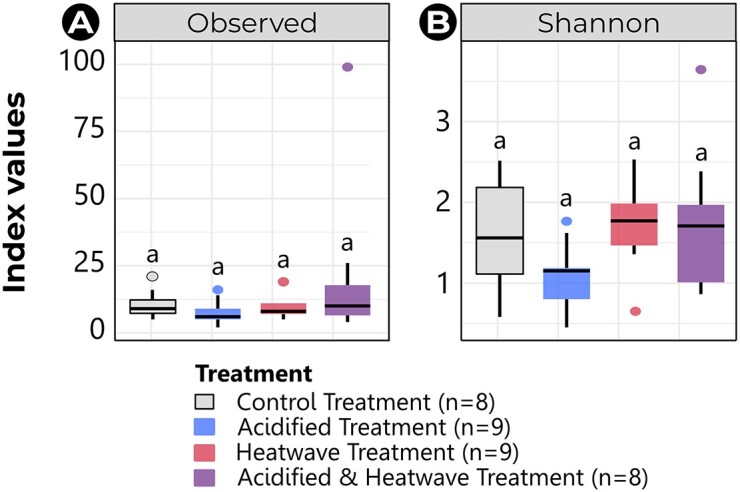
Alpha diversity indexes (within-sample diversity) of the NM in Atlantic cod reared under climate change-related treatments, as measured by the observed richness (A) and the Shannon diversity index (B). Boxes and dots are coloured by treatment. Treatments sharing the same letter are not significantly different (*p*-adj > .05). Note: Tromsø seawater samples did not meet DNA quality requirements.

A total of 365 ASVs were identified in the cod rosette samples distributed across different treatments: 70 in CT, 53 in AT, 77 in HT, and 165 in AHT. Notably, the rosette sample ‘CC232’ from AHT alone accounted for 99 ASVs. None of the indexes revealed significant differences in cod NM diversity between tanks (*p*-adj = 1, Table S2). Therefore, samples from the triplicated tanks were merged within treatment. Similarly, observed richness (mean ± SD: CT = 10.5 ± 5.5, AT = 7.7 ± 4.7, HT = 10.0 ± 5.4, and AHT = 22 ± 31.9; *p*-adj = 1; Fig. 5A) and Shannon diversity indexes (CT = 1.6 ± 0.7, AT = 1.1 ± 0.4, HT = 1.8 ± 0.6, and AHT = 1.8 ± 0.9; *p*-adj = 1; Fig. 5B) of cod NM did not show any significant difference according to treatment (Table S2).

#### Beta diversity

**Figure 6.**
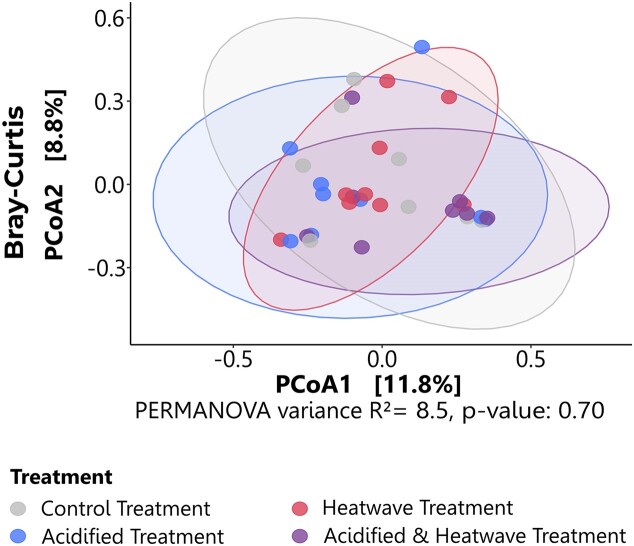
PCoA illustrating beta diversity of the NM in Atlantic cod reared under climate change-related treatments. PCoAs were computed on Bray–Curtis dissimilarity matrices. Each dot represents a sample and is coloured by treatment.

Dissimilarities using either distance method explained <9% of data variability and did not vary with treatment (p_Bray–Curtis_ = 0.70; p_Wunifrac_ = 0.76) (Fig. 6, Table S3, see also for Jaccard and Unifrac results). Although PCoA showed clustering of rosette samples according to treatment, clusters overlap substantially (Fig. 6).

#### Taxonomic profiles

**Figure 7.**
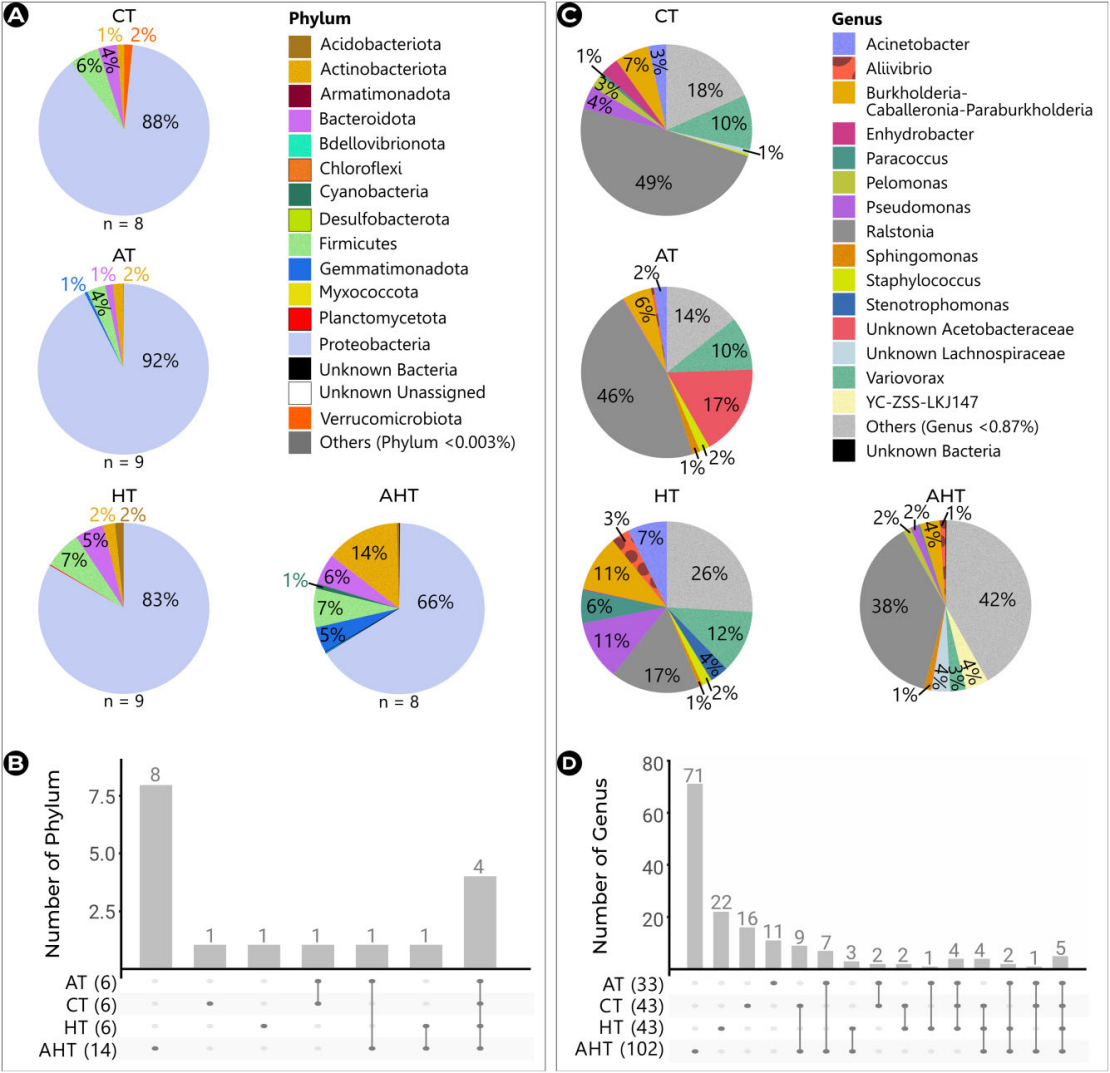
Taxonomic profile of the NM in Atlantic cod reared under climate change-related treatments. Proportional distribution of the 15 most predominant bacterial phyla (A) and genera (C) including the number of exclusive and common phyla (B) or genera (D). The total number of phyla or genera per condition is in parenthesis. CT: Control Treatment, AT: Acidified Treatment, HT: Heatwave Treatment, and AHT: Acidified and Heatwave Treatment.

Cod NM exposed to climate change-related treatments comprised a total of 15 identified phyla and 123 identified genera. Among these, 6 bacterial phyla were found in CT, 6 in AT, 6 in HT, and 14 in AHT, respectively distributed in 43, 33, 43, and 102 genera (Fig. 7).

Treatment did not impact the abundance of any phylum. Four phyla consistently dominated cod NM across all treatments, listed in decreasing order: *Proteobacteria* (ranging from 66% to 92%), *Actinobacteriota* (1%–14%), *Firmicutes* (4%–7%), and *Bacteroidota* (1%–6%) (Fig. 7A). The greatest richness of exclusive phyla [*Cyanobacteria, Armatimonadota, Bdellovibrionota, Chloroflexi, Dependentiae* (currently *Candidatus Babelota*), *Desulfobacterota* (currently *Pseudomonadota*), *Myxococcota*, and Unknown Bacteria] was observed in AHT (Fig. 7B). CT and HT each displayed one exclusive phylum, *Verrucomicrobiota* (relative abundance: 2%) for CT and *Planctomycetota* (relative abundance: 0.2%) for HT. In contrast, no exclusive phylum was identified in AT (Fig. 7B). An unknown unassigned phylum was present in the control temperature treatments (at 0.008% in CT, and 0.1% in AT), while the phylum *Gemmatimonadota* was shared by both acidified treatments (1% in AT, 5% in AHT) and the phylum *Acidobacteriota* was shared by both heatwave treatments (2% in HT, 0.4% in AHT) (Fig. 7A).

Four genera, belonging all to *Proteobacteria* (*Pseudomonas, Burkholderia–Caballeronia–Paraburkholderia, Variovorax*, and *Ralstonia)* were consistently present across all treatments (Fig. 7C). Notably, *Ralstonia* dominated, exhibiting abundances between 17% and 49% (Fig. 7C). Treatment exclusivity was observed for numerous genera, with the highest number (71) identified in AHT (Fig. 7D). Interestingly, only *Pseudomonas* displayed a statistically significant difference in abundance between treatments. It was 5 LFC more abundant in HT rosettes (43%, *p*-adj_ANCOM_ = .03) compared to AT rosettes (0.2%) (Fig. S2D).

As for the species comparison analysis, high interindividual variability was also evident in the climate change-related simulation on cod. For example, *Pseudomonas* abundance varied considerably. It was present in only one AT sample (1.2%) but found in seven HT samples (1.6%–37%), two AHT samples (2.2%–10.3%), and four CT samples (5%–55%). Similarly, the dominant *Ralstonia* was present in 35% of all samples with abundances ranging from 0.4% to 84% (Fig. S4).

#### Beta dispersion

Interindividual variability of cod NM based on Bray–Curtis beta dispersion did not significantly vary with treatment (Permutation test F = 0.60, *p*-adj_Bray–Curtis_ = .6), with dispersion distances from the centroid almost identical across treatments (DispDist_CT_ = 0.61, DispDist_AT_ = 0.57, DispDist_HT_ = 0.63, and DispDist_AHT_ = 0.62). However, based on Wunifrac beta dispersion (Permutation test F = 2.92, *p*-adj_Wunifrac_ = .04), there was a significantly lower DispDist in the control (DispDist_CT_ = 0.06) than in the other treatments (DispDist_AT_ = 0.11, DispDist_HT_ = 0.10, and DispDist_AHT_ = 0.14) (Table S4).

## Discussion

We present here the first characterization of the NM of two marine fish species, the European seabass (*D. labrax* from Brest and Palavas), and the Atlantic cod (*G. morhua* from Tromsø) reared in captivity under ambient conditions. We provide a comparative analysis of the taxonomic profile and the community structure of the NM in these commercially and socio-economically important top predators. Additionally, we established a baseline understanding of interindividual variability within each group based on a model built on a significant number of specimens (*n* = 32–38). Furthermore, we offer the first insights into the impacts of acidification and a simplified heatwave on the taxonomic profile and community structure of the cod NM reared under two different climate change-related stressors, and the combination of them, for 31 days.

### General structure of the NM

The NM of both seabass and cod was predominantly composed of *Proteobacteria*, followed by *Actinobacteriota, Bacteroidota*, and *Firmicutes* for seabass. However, in cod, *Firmicutes* was the second or third most abundant phylum after *Proteobacteria*, both under ambient and climate change-related conditions. The same phyla, notably *Proteobacteria*, also dominated the NM in other vertebrate species in the few known studies to date. This includes mammals such as cotton rats (Chaves-Moreno et al. 2015), mice (François et al. 2016, Casadei et al. 2019), four species of tortoises (Weitzman et al. 2018), the freshwater zebrafish (Casadei et al. 2019), and the anadromous rainbow trout (Lowrey et al. 2015). As expected, the relative abundances of these phyla and the overall taxonomic profile at lower taxonomic levels vary across species. While the two prior studies investigating fish NM composition provide valuable insights for comparisons across diverse habitats and host physiology, they are limited to the phylum level, preventing genus-level comparisons.

The relative abundance of *Proteobacteria* was highest in the freshwater zebrafish (92%–98%, *n*=6; Casadei et al. 2019) and lowest in the anadromous rainbow trout (38%–65%, *n*=5; Lowrey et al. 2015). In our study, the abundance of *Proteobacteria* present in the NM of all fish groups varied widely between individuals ranging from 13% to 100% (*n* = 34) in Brest seabass, from 80% to 98% (*n* = 32) in Palavas seabass, and from 8% to 100% (*n* = 38) in Tromsø cod reared in ambient conditions. Although, *Proteobacteria* abundance also varied within treatments in cod NM, the range of abundances was narrower in most climate change-related conditions compared to ambient conditions: 53%–100% in the Control Treatment (*n* = 8), 0%–100% in the Acidified Treatment (*n* = 9), 48%–100% in the Heatwave Treatment (*n* = 9), and 28%–99% in the Acidified and Heatwave Treatment (*n* = 8). The variability observed in our study exceeded previously reported levels across the vertebrate species studied to date, with the exception of zebrafish, which exhibited near-identical abundance across samples with an overabundance of *Proteobacteria*. However, it is important to consider that sample sizes might partially explain the observed differences in variability.

Consistent with observations in other marine fish, this study found *Proteobacteria* to be the dominant phylum across all previously investigated microbiota compartments (rosette, skin, gill, and gut). However, the relative abundance of *Proteobacteria* and the other dominant phyla (*Actinobacteriota, Bacteroidota*, and *Firmicutes*) varied between microbiota compartment within a fish species (Llewellyn et al. 2014, Lowrey et al. 2015). For instance, after *Proteobacteria, Bacteroidota* was the dominant phyla in skin and gill microbiota (Pimentel et al. 2017, Rosado et al. 2019a), whereas *Firmicutes* dominated the gut microbiota in seabass (Serra et al. 2021, Rangel et al. 2022). These variations likely reflect the specific ecological niches and functions associated with each body organ. Microbiota structure can be further modulated by diet (Serra et al. 2021, Rangel et al. 2022), geographic location (Walter et al. 2019), and season (Larsen et al. 2015) at both the individual level and between fish species (Chiarello et al. 2015).

At the genus level, the NM of both seabass and cod largely differed from previously studied fish species. In zebrafish, the NM was dominated by an unidentified genus from the *Aeromonadaceae* family (25%) belonging to *Proteobacteria* (Casadei et al. 2019). The high abundance of *Aeromonadaceae* in zebrafish NM and the practical absence in seabass and cod NM, likely reflects an adaptation to their freshwater habitat. Indeed, some genera within this family are known to thrive in low-salinity environments and are rare in the marine environment (Fischer-Romero et al. 1996, Fernández-Bravo and Figueras 2020). The observed variations in the NM taxonomic profile between fish species could be partially attributed to physiological adaptations driven by the physicochemical parameters of their respective habitats. In contrast to zebrafish, which are intolerant to high-salt environments, seabass and cod are adapted to tolerate the high salinity of open waters and to venture into lower salinity conditions found in estuaries (Kijewska et al. 2016).

Further strengthening the link between NM composition and environmental factors, *Stenotrophomonas* was virtually absent in zebrafish NM (0.2%; Casadei et al. 2019) and Tromsø cod (0.6%), while it dominated the seabass NM (32% in Brest and 76% in Palavas). These contrasting abundances suggest the influence of factors beyond salinity. Notably, *Stenotrophomonas* spp. exhibit a broad ecological niche, thriving in diverse environments such as food, soil, plant rhizospheres, freshwater and seawater, with optimal growth at temperatures between 22°C and 30°C (Romanenko et al. 2008, Mahdi et al. 2014, Urase et al. 2023). Consistent with this, the highest abundance of *Stenotrophomonas* in the warmer Palavas seawater (22.5°C) compared to the moderate abundance in the temperate Brest seawater (11.6°C) supports the hypothesis that habitat characteristics, particularly temperature, may influence the abundance of specific bacterial taxa within the fish NM.

Recent investigations have demonstrated that host age can significantly impact the fish microbiota. Rosado et al. (2021) reported a notable increase in alpha diversity of the skin microbiota in older seabass compared to younger individuals. In contrast, no significant age-related differences were observed in the alpha diversity of the gill microbiota in seabass or the skin, gill, or gut microbiota of seabream (Piazzon et al. 2019, Rosado et al. 2021). However, most beta diversity traits did differ in all of these microbiota types and fish species (Piazzon et al. 2019, Rosado et al. 2021). These findings suggest that the influence of age on the microbiota is contingent upon both the specific anatomical compartment and the fish species. Taken together, these findings highlight the complex interplay between environmental factors and host traits in shaping the fish microbiota diversity and structure.

Surprisingly, several unresolved taxonomies, including an unknown phylum (Unknown Bacteria) were observed in this study. This highlights the prospect for discovering novel symbiotic relationships between fish and bacteria and offers the potential to elucidate a diverse array of metabolisms and symbiotic functionalities. For instance, *Stenotrophomonas* spp. and *Variovorax* spp. are ubiquitous in natural environments, including marine habitats, and in symbioses with plants (Satola et al. 2013, Urase et al. 2023). *Stenotrophomonas* spp. are also found in symbiosis with deep-sea invertebrates and as an opportunistic pathogen resistant to antibiotics in clinical materials (Romanenko et al. 2008, Urase et al. 2023). Due to their wide range of metabolic properties, including the resistance to heavy metals and the ability to degrade a variety of pollutants, both genera are suitable candidates for bioremediation and phytoremediation applications in biotechnology (Ryan et al. 2009, Sun et al. 2018). Furthermore, they both are able to produce plant-protective antimicrobial substances and plant growth-promoting factors (Ryan et al. 2009, Sun et al. 2018). Intriguingly, these functionalities translate to beneficial interactions between bacteria and their plant hosts raising the question: could these genera offer similar benefits to their fish hosts? Further research is needed to explore the ecological roles of the NM and its potential contribution to fish physiology and health.

### Comparative analysis of the NM in two marine fish species and interindividual variability

The structure of the NM community exhibited variation both within and between-species. Interestingly, while the NM of seabass from Brest and Palavas displayed similar richness, the strong dominance of *Stenotrophomonas* in Palavas seabass NM resulted in reduced evenness (abundance distribution) and consequently, lower alpha (within-sample) and beta (between-sample) diversity. This indicates that the NM in seabass has a phylogenetically close profile, but the relative abundance of taxa explains the within-species dissimilarities in the seabass NM across different habitats (Brest and Palavas). On the other hand, abundance and phylogeny together seem to be a more important driver in determining between-species dissimilarities. The significantly higher abundance of *Variovorax, Burkholderia–Caballeronia–Paraburkholderia*, and the greater proportion of rare genera in Tromsø cod NM, likely contributed to enlarge the differences in community structure between fish species.

Concerning interindividual variability within each microbial community, Palavas seabass NM exhibited minimal community structure variability between samples whether based on abundance alone or on combined abundance and phylogeny. This aligns with our Interindividual Variability Model, suggesting that the smallest sample size of 8–12 samples is sufficient to capture 95%–99% of the variation in the Palavas seabass NM. Conversely, the wider sample size range for Tromsø cod NM (13–25 samples) and Brest seabass NM (9–20 samples) reflects a greater fluctuation in mean DispDist predicted by our model. For future experimental testing of hypotheses, our findings suggest that large sample sizes are necessary to cover interindividual variability, notably for Tromsø cod. This will minimize potential bias and ensure robust conclusions. We acknowledge the potential challenges associated with collecting large sample sizes (ethical considerations, cost, or logistical limitations), particularly in replicated experiments. Therefore, we recommend careful consideration when selecting a sample size within the proposed ranges while considering the aim of the trial, appropriate experimental design and statistical approaches to minimize sample size requirements while maximizing statistical robustness. Nevertheless, we strongly advise against using less than four samples per biological replicate, as variation (DiffVar) can reach up to 33% in such scenarios (refer to Table S5 for details). It is noteworthy to highlight that a portion of this variability may be attributable to other random parameters, such as technical variations during the processes of rosette dissection and DNA extraction, technical ghost factors in laboratory experiments (Galloway et al. 2020) together with different farming practices and specific antibiotic and probiotic use (Pimentel et al. 2017, Rosado et al. 2019b). All these aspects, particularly the use of antibiotics. However, accurately quantifying the random contributions of these factors to variability remains challenging.

### Seabass NM specificity regarding seawater bacterial community

As generally observed by comparing fish microbiota and seawater diversity (e.g. Sadeghi et al. 2023), alpha diversity metric revealed a significantly higher bacterial diversity in seawater compared to seabass NM, with seawater exhibiting nearly double the diversity. However, this statement must be made with caution considering the difference in sample size and biomass between rosettes and seawater samples. Indeed, while differences in sampled bacterial biomass, could contribute to variations in alpha diversity (Padilla et al. 2015), the consistent sampling and filtering protocols across Brest and Palavas suggest that this factor alone is unlikely to fully account for significant differences in alpha diversity only observed in Brest. Brest seawater harboured a richer bacterial community, encompassing 24 phyla distributed across 199 genera, compared to the 11 phyla and 106 genera identified in the Brest seabass NM. Beta diversity analysis, focusing solely on abundance data, revealed significant differences between the bacterial communities of seawater and the NM for both Brest and Palavas. This finding suggests distinct community structures between the surrounding seawater and the fish NM. The presence of all dominant bacterial genera in seabass rosettes within the seawater suggests that at least a portion of the NM may originate from the surrounding environment as previously observed in salmon gut microbiota (Dehler et al. 2017).

While the nasal environment appears to support the growth of various genera, not all prevalent seawater bacteria readily colonized the NM. Some genera were significantly more abundant in either seawater or the NM, highlighting that different selective pressures or specific adaptations are required for thriving within the nasal environment. Furthermore, the genera *Amylibacter, Candidatus_Puniceispirillum*, and SAR86_clade were exclusively present in seawater and absent in seabass NM, and even absent in other previously studied microbiota in seabass such as gut, skin, or gills (Rosado et al. 2019a, Serra et al. 2021). This confirms that some genera are unlikely to colonize the epithelium of fish despite their presence in seawater. Diet, endogenous physiology, and immunological state of the host (Apprill et al. 2014, Weitzman et al. 2018, Rosado et al. 2019b, Torrecillas et al. 2023) together with bacterial physiology play a role in limiting or facilitating the colonization of some genera. Furthermore, the rearing system type (flow-through in Brest versus semiopen in Palavas), the rearing tanks cleaning methods, the seawater filtration and rosette sampling and DNA extraction methods, and the emergence of biofilm in the tanks could influence the seawater’s phytoplanktonic and bacterial communities. However, definitively attributing the observed differences in specific bacterial genera solely to seawater parameters, rearing system characteristics, or a combination of both remains challenging due to their inherent confounding nature.

### First insights on the impacts of acidification and a simplified heatwave in the community structure of cod NM

The taxonomic profile and Shannon diversity values of the NM in Tromsø cod exposed to acidification and a simplified heatwave, either alone or in combination, displayed minimal variation compared to control conditions. This suggests that the overall diversity of the cod NM was relatively unaffected by the implemented treatments, as confirmed by alpha and beta diversity. However, the taxonomic profile of cod NM exposed to the combination of acidification and heatwave (AHT) appeared different from that of the Control Treatment (CT), though this was not supported statistically. Notably, AHT-exposed cod exhibited the highest richness, harbouring 14 phyla and 14 genera compared to only 6 phyla and 8 genera in CT cod. At the phylum level, *Actino*bacteriota and *Gemmatimonadota* were more abundant in AHT compared to CT. Similarly, at the genus level *Acinetobacter, Sphingomonas*, and *Variovorax* displayed variations in relative abundance between AHT and CT cod.

While beta diversity analysis revealed no statistically significant impact of treatment on Tromsø cod NM, beta dispersion based on abundance and phylogeny was significantly higher in every treatment compared to CT (DispDist_CT_ = 0.06, DispDist_AT_ = 0.11, DispDist_HT_ = 0.10, and DispDist_AHT_ = 0.14), indicative of a higher level of data variability after exposure. In addition, PCoAs displayed some degree of treatment-related clustering. The phylum *Acidobacteriota* was exclusive to both heatwave treatments, while *Gemmatimonadota* was solely found in acidified treatments. This suggests a potential for subtle shifts in community structure between treatments. However, these PCoA analyses explained <9% of the observed variation, indicating that other factors likely play a more substantial role in shaping the cod NM composition.

A recent study showed that coastal cod exhibit higher physiological tolerance to climate change-related stressors (warming, acidification, and freshening) compared to offshore populations, due to genotypic differences (Perry et al. 2024). Although this might poorly explain the lack of significant diversity differences between treatments, it is yet uncertain whether this increased tolerance is maintained in captive-bred lineages such as the cod used in our study. In an earlier study, the intestinal microbiota in wild-caught Atlantic cod were not affected by captive rearing for 6 weeks, however its diversity was reduced by artificial feeding (Dhanasiri et al. 2011). Furthermore, understanding the mechanisms linking increased climate change physiological tolerance to NM resilience requires further investigation. This includes exploring the role of other physiological traits, such as immune response, and elucidating the complex crosstalk between the immune system and NM (Yu et al. 2021).

While the lack of significant alpha and beta diversity differences between treatments may have multiple contributing factors, it can be most likely primarily attributable to methodological limitations. These limitations deserve to be highlighted to be carefully addressed in future experimental designs. To begin with the high interindividual variability observed in Tromsø cod NM. Beta dispersion analysis identified Tromsø cod as the most variable group compared to Brest and Palavas seabass, requiring a larger sample size (13–29 individuals) to capture 95%–99% of the variability according to our model. To address this, we opted to pool the triplicated tanks per treatment, increasing the sample size per treatment despite potential pseudo-replication concerns. This decision aligns with previous findings on the influence of sample size in analysing microbiomes (Knight et al. 2018). Pooling resulted in eight to nine samples per treatment, predicted to cover 96% of the variability. While a coverage of 96% is reasonable, care should be taken when asserting that the variability has been properly covered. Indeed, our model was based on cod reared under ambient conditions, though exposure to acidification and/or the simplified heatwave significantly increased variability compared to the control as shown by beta dispersion (DispDist).

The influence of the flow-through system on the seawater bacterial community cannot be disregarded. Since the seawater itself did not experience the same prolonged heat and pH stress as the fish, the lack of significant NM variations across treatments might partially be explained by the seawater community’s limited time to respond. However, the absence of Tromsø seawater samples precludes a definitive confirmation or rejection of this hypothesis. Another factor potentially influencing the absence of a treatment effect is the sampling time point. Rosettes were collected 6 days after the simplified heatwave, when temperatures had returned to control levels (8°C). We cannot determine whether the peak temperature (16°C) enhanced or hindered bacterial growth and colonization within the NM, and given the very limited knowledge of this microbiota, a return to the previous equilibrium after stress cannot be excluded. The rapid life cycles of some bacteria suggest that 6 days at a lower temperature may have been sufficient to reset growth to basal levels. This observation might indicate a degree of resilience in the NM to rapid and acute changes in seawater temperature and pH.

To definitively assess the impact of heatwave events on Tromsø cod NM plasticity, further experiments are necessary. These studies should employ a larger sample size with both biological replicates (independent fish) and technical replicates (repeated sampling from the same fish), as well as replicated seawater samples, at multiple phases of a heatwave, and within a recirculating system. This comprehensive approach would allow for a more robust understanding of the dynamics within the NM throughout the heatwave cycle in a more controlled experimentation of the effects of environmental changes on the seawater community and its subsequent influence on fish NM. While logistical limitations and ethical considerations might arise with larger sample sizes, these can be addressed through careful experimental design and adherence to animal welfare protocols. Ultimately, such research will be crucial to determine the resilience of the cod NM in the face of increasingly frequent and severe heatwave events associated with climate change.

## Conclusion

The present study characterizes for the first time the NM in two strictly marine fish species, European seabass and Atlantic cod, reared under ambient conditions. Our findings suggest a distinct NM composition compared to previously studied freshwater and anadromous fish species. Furthermore, the NM structure differed significantly between seabass and cod, suggesting potential adaptations to their respective ecological niches. While interindividual variability was high, seabass displayed a core NM consisting of *Citrobacter, Elizabethkingia, Enterobacter, Rhodococcus*, and *Stenotrophomonas*, whereas cod’s core NM solely comprised *Burkholderia–Caballeronia–Paraburkholderia* and *Variovorax*. Within-species variation in seabass NM was observed between sites (Brest and Palavas) and driven primarily by relative abundance variations, not overall phylogenetic profiles. This highlights the significant role of environmental factors, such as habitat variations, in shaping the NM beyond fish species. The presence in seawater of all of the most abundant genera of seabass NM suggests an environmental origin for at least a portion of the NM. However, the absence of some seawater genera in the NM implies selective colonization by specific bacterial taxa. The structure of cod NM remained unaffected by the simulated climate change-related scenarios. This might be attributed to multiple methodological limitations including small sample size, potentially failing to capture the full extent of interindividual variability. Our model suggests a minimum of 13 samples per replicate for cod’s NM studies, which may need to be further increased to capture enhanced variability due to exposure to climate change-related stressors. Nevertheless, our study provides a valuable foundation for future research on how environmental factors modulate the NM of marine fish.

## Supplementary Material

fiaf018_Supplemental_Files
