# Supplementary material for: The nasal microbiota of two marine fish species: diversity, community structure, variability, and first insights into the impacts of climate change-related stressors
Source: FEMS Microbiol Ecol. 2025 Feb 17;101(3):fiaf018. doi: 10.1093/femsec/fiaf018 (PMC11879562; doi:10.1093/femsec/fiaf018)
Supplement: fiaf018_Supplemental_Files [file fiaf018_supplemental_files.zip › Supplementary Figure Legendsv2.docx]

**Supplementary Figure Legends**

**Figure S1.** Simplified scheme showing the experimental setup (**A**) and treatments timeline across the different phases (**B**) of the rearing of Atlantic cod *Gadus morhua* exposed to four climate change-related treatments.

**Figure S2.** Sampling location of the nasal microbiota in Brest seabass *Dicentrarchus labrax*. (**A**) Rostrum section of an adult seabass (7-year-old), highlighting the olfactory rosette (pink donut) as the target sampling area for total DNA extraction. (**B**) Scanning Electron Microscope (SEM) micrograph of a juvenile seabass rosette (1 year-old). The rosette is visibly covered by a layer of mucous membrane.

**Figure S3.** Analysis of Composition of Biomes (ANCOM) for the most predominant genera according to rosette site (**A**), or to seawater site (**B**), or to both rosette and seawater site (**C**) for the species comparison analysis, or according to treatment for the climate change-related simulation (**D**). Comparisons between groups with significantly different log fold change (LFC) are shown. HT: Heatwave Treatment, AT: Acidified Treatment. Comparisons’ significances are shown as: * if 0.01 < p-adj ≤ 0.05, ** if 0.001 < p-adj ≤ 0.01, *** if p-adj ≤ 0.001.

**Figure S4**. Relative abundance of the most predominant genera present in each sample of seabass or cod rosette and seawater from Brest (**A**), Palavas (**B**) or Tromsø (**C**).

**Figure S5**. Relative abundance of the most predominant genera present in each sample of Tromsø cod rosette according to treatment. CT: Control Treatment, AT: Acidified Treatment, HT: Heatwave Treatment and AHT: Acidified & Heatwave Treatment.
